# Digging deeper: structural background of PEGylated fibrin gels in cell migration and lumenogenesis†

**DOI:** 10.1039/c9ra08169k

**Published:** 2020-01-24

**Authors:** A. I. Shpichka, P. V. Konarev, Yu. M. Efremov, A. E. Kryukova, N. A. Aksenova, S. L. Kotova, A. A. Frolova, N. V. Kosheleva, O. M. Zhigalina, V. I. Yusupov, D. N. Khmelenin, A. Koroleva, V. V. Volkov, V. E. Asadchikov, P. S. Timashev

**Affiliations:** Institute for Regenerative Medicine, Sechenov University 2-8 Trubetskaya St. Moscow Russia 119991 ana-shpichka@yandex.ru yu.efremov@gmail.com naksenova@mail.ru slkotova@mail.ru nastyfr@ya.ru timashev.peter@gmail.com +7 495 6091400 ext. 3638; A. V. Shubnikov Institute of Crystallography, Federal Scientific Research Center “Crystallography and Photonics” RAS Moscow Russia peter_konarev@mail.ru krukovaae@yandex.ru zhigal@crys.ras.ru dirq@rambler.ru volkicras@mail.ru asad@crys.ras.ru; National Research Center “Kurchatov Institute” Moscow Russia; Department of Polymers and Composites, N. N. Semenov Institute of Chemical Physics Moscow Russia; FSBSI ‘Institute of General Pathology and Pathophysiology’ Moscow Russia; Faculty of Biology, Lomonosov Moscow State University Moscow Russia; Bauman Moscow State Technical University Moscow Russia; Institute of Photon Technologies, Federal Scientific Research Center “Crystallography and Photonics” RAS Moscow Russia iouss@yandex.ru; Laser Zentrum Hannover e. V. Hannover Germany info.koroleva@gmail.com

## Abstract

Fibrin is a well-known tool in tissue engineering, but the structure of its modifications created to improve its properties remains undiscussed despite its importance, *e.g.* in designing biomaterials that ensure cell migration and lumenogenesis. We sought to uncover the structural aspects of PEGylated fibrin hydrogels shown to contribute to angiogenesis. The analysis of the small-angle X-ray scattering (SAXS) data and *ab initio* modeling revealed that the PEGylation of fibrinogen led to the formation of oligomeric species, which are larger at a higher PEG : fibrinogen molar ratio. The improvement of optical properties was provided by the decrease in aggregates' sizes and also by retaining the bound water. Compared to the native fibrin, the structure of the 5 : 1 PEGylated fibrin gel consisted of homogenously distributed flexible fibrils with a smaller space between them. Moreover, as arginylglycylaspartic acid (RGD) sites may be partly bound to PEG-NHS or masked because of the oligomerization, the number of adhesion sites may be slightly reduced that may provide the better cell migration and formation of continuous capillary-like structures.

## Abstract 2

Fibrin is a well-known tool in tissue engineering, but the structure of its modifications created to improve its properties remains undiscussed despite their importance, *e.g.* in designing biomaterials that ensure cell migration and lumenogenesis.

## Graphical abstract

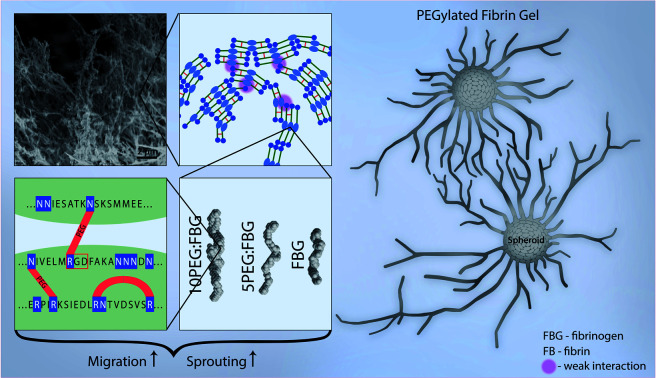

## Introduction

One of the critical issues in new organ and tissue fabrication is vascularization that is usually caused by cell migration and lumenogenesis and ensures the nutrient and oxygen supply and metabolite removal. Despite the progress achieved, most studies are based on spontaneous vessel formation within tissue-engineered constructs.^1–3^ In such cases, the formed microvasculature is highly random and cannot provide a sufficient supply of nutrients and oxygen because of the lack of anastomoses and large spacing between vessels. Therefore, one should guide the angiogenesis within newly fabricated tissues that can be achieved *via* biochemical and mechanical cues provided by the microenvironment.^4^

In tissue engineering, the microenvironment is mostly formed by biomaterials (scaffolds, hydrogels, *etc.*). Among them, fibrin has been shown to be an effective tool to produce capillary-like networks and can be used as a biomaterial design platform to fabricate pre-vascularized tissues.^5–9^ Fibrin is forme *via* the thrombin-associated cleavage of fibrinogen, a blood plasma protein, followed by its polymerization.^10^ Natural fibrin rapidly degrades and is not transparent, so several modifications have been suggested to overcome these limitations and, moreover, those have been shown to increase its angiogenic potential.^11–13^ Among them, PEGylation (modification with functionalized polyethylene glycol (PEG)) is of particular interest.

Previously, it was shown that PEGylation allowed not only achieving the gel transparency and better stability, but also improving its angiogenic properties (*e.g.* they supported the formation of lumens).^11,14,15^ However, the structural aspects remain undiscussed despite their evident importance in designing angiogenesis-guiding biomaterials. Earlier, we determined the shape of fibrinogen in solution using small angle X-ray scattering (SAXS).^16^ Frisman *et al.*^17^ investigated the structural properties of PEG–fibrinogen conjugates formed *via* a Michael-type addition of thiols to acrylate-functionalized PEG. However, the latter procedure is time- and labor-consuming and requires protein refolding that is hard to be controlled and causes significant changes in the protein native structure. To avoid the aforementioned problems, another common type of fibrin PEGylation using NHS-functionalized PEG was studied by several groups.^12,15,18–21^ This type of modification proceeding through the covalent binding of amino groups does not require protein refolding and is easy to carry out. However, despite the good results in biological experiments, no study was dedicated to the structural properties of this modification, though this knowledge could help in understanding cell–matrix interactions and possible ways to tune them. In this study, we therefore set the goal to reveal the structural aspects of the PEGylated fibrin hydrogel that provide a favorable environment to cells and may facilitate the angiogenesis.

## Results

### FT-IR spectral analysis

**Fig. 1:**
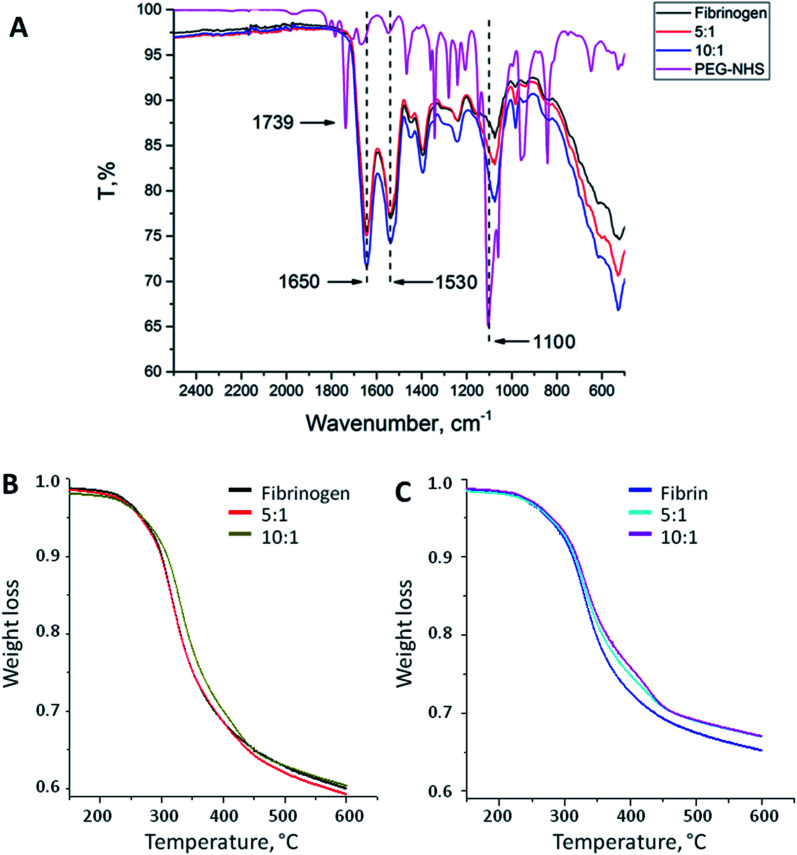
(A) FT-IR spectra of native fibrinogen, PEG–fibrinogen conjugates (5 : 1 and 10 : 1), and PEG-NHS. The PEGylated fibrinogen samples had greater peaks at 1100 cm^−1^ that revealed the insertion of PEG-derived (C–O) units. Amide I and amide II bands (1650 and 1530 cm^−1^) typical for PEG-NHS were noticed in the spectra of the fibrinogen modifications. (B) Thermal destruction of native fibrinogen and its modifications. (C) Thermal destruction of native fibrin and its modifications.

PEG binding to the fibrinogen backbone was proven by FT-IR spectra. Fig. 1A shows the corresponding spectra of NHS-functionalized PEG and native and PEGylated (5 : 1, 10 : 1) fibrinogen. Succinimidyl ester triplet band (1739, 1782, and 1813 cm^−1^) is specific for PEG-NHS; it was not observed in the modified fibrinogen spectra because of the reaction with primary amino groups followed by imide ring opening. Samples of the fibrinogen modifications had an increased intensity band at 1100 cm^−1^ that proves the PEG-derived (C–O) units insertion. This band varies depending on the molar ratio. The same peaks as those in the PEG-NHS spectrum for amide I and amide II bands (1650 and 1530 cm^−1^) were noticed in the spectra of the PEGylated fibrinogen samples. The increased C–O units and amide peaks proved the successful PEG insertion into the fibrinogen backbone.

### Thermal gravimetry analysis

Fig. 1B and C show the temperature dependences of the mass loss (thermal gravimetry, TG) acquired during the thermal destruction (TD) of native and PEGylated fibrinogen (B) and native and PEGylated fibrin (C). Fibrin samples were more thermally stable than fibrinogen: there were small shifts (app. 20 °C) to the high-temperature region of the initial mass loss temperature on the TG curves. This does not appear surprising because fibrin is a polymer representing monomer chains within fibrinogen which crosslink after thrombin-associated cleavage. On the TG curves, there is a region with a small mass loss that may be associated with the carbon residue formation. The carbon residue content was different: for the fibrin samples, it was app. 10% higher than that for the fibrinogen samples that was the evidence for the successful fibrin cross-linking. The PEGylated fibrin samples had a slightly higher carbon residue content than native fibrin samples had that presumably confirmed a higher cross-linking degree of the PEGylated fibrin. However the small amount of PEG in those samples might have resulted in such difference of the TG curves, as well.

### Turbidity analysis

**Fig. 2:**
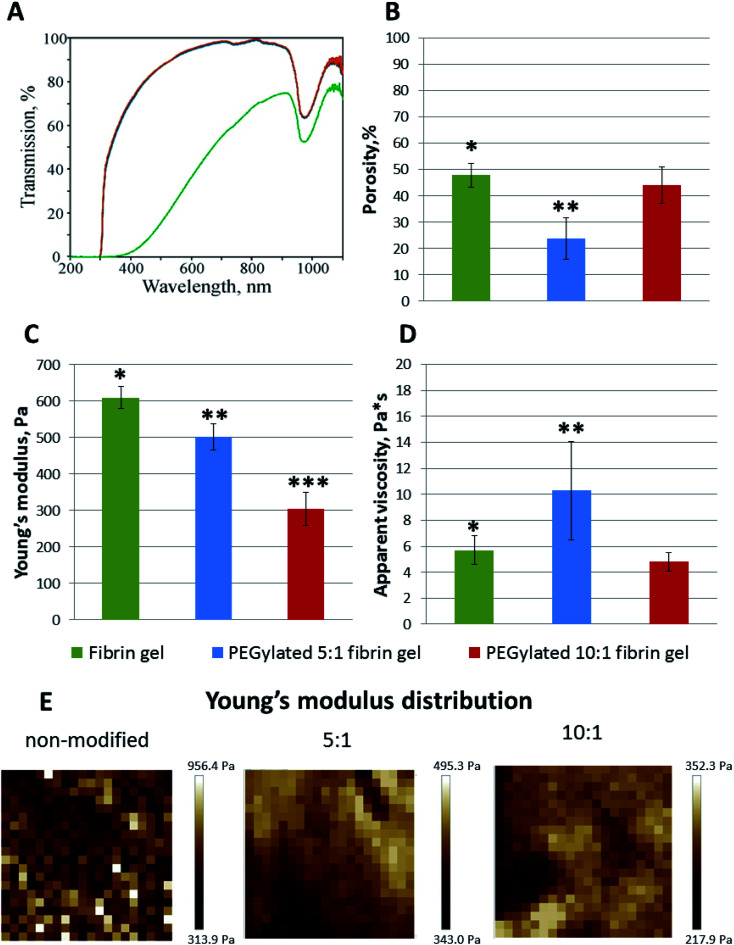
(A) Optical properties of native and modified fibrin gels (UV-Vis transmission spectra, green – non-modified, blue – 5 : 1 PEGylated, red – 10 : 1 PEGylated fibrin gels). Compared to the non-modified fibrin, the visible light transmission through the PEGylated fibrin gels was significantly enhanced. (B) Porosity of native and modified fibrin gels, mean ± SD. (C)–(E) Mechanical properties of gels: Young's modulus (Pa), mean ± SD (C), and its distribution maps (E); apparent viscosity (Pa s), mean ± SD (D). Young's moduli were slightly inhomogeneous for the PEGylated fibrin gels and more heterogeneous for the non-modified gel, in which regions with significantly higher Young's moduli were observed. PEGylation led to a decrease of the Young's modulus. * Fibrin gel *vs.* PEGylated 5 : 1 fibrin gel, *p* < 0.05; ** PEGylated 5 : 1 fibrin gel *vs.* PEGylated 10 : 1 fibrin gel, *p* < 0.05; *** PEGylated 10 : 1 fibrin gel *vs.* fibrin gel, *p* < 0.05.

The samples prepared from 10 : 1 and 5 : 1 PEGylated fibrinogen were transparent whereas the gels from native fibrinogen became turbid upon thrombin crosslinking (Fig. 2A). The visible light transmission through the modified fibrin gels was significantly increased compared to that for the native gel: 66% at a wavelength of 380 nm and 98% – at 780 nm (*vs.* 1% and 63%, respectively, for the native fibrin gel) (Fig. 2A). Both modifications had almost the same absorbance spectra (Fig. 2A). The observed peak in a range of 900–1000 nm (Fig. 2A) corresponded to the well-known absorbance peak of water.

### Confocal laser scanning microscopy

**Fig. 3:**
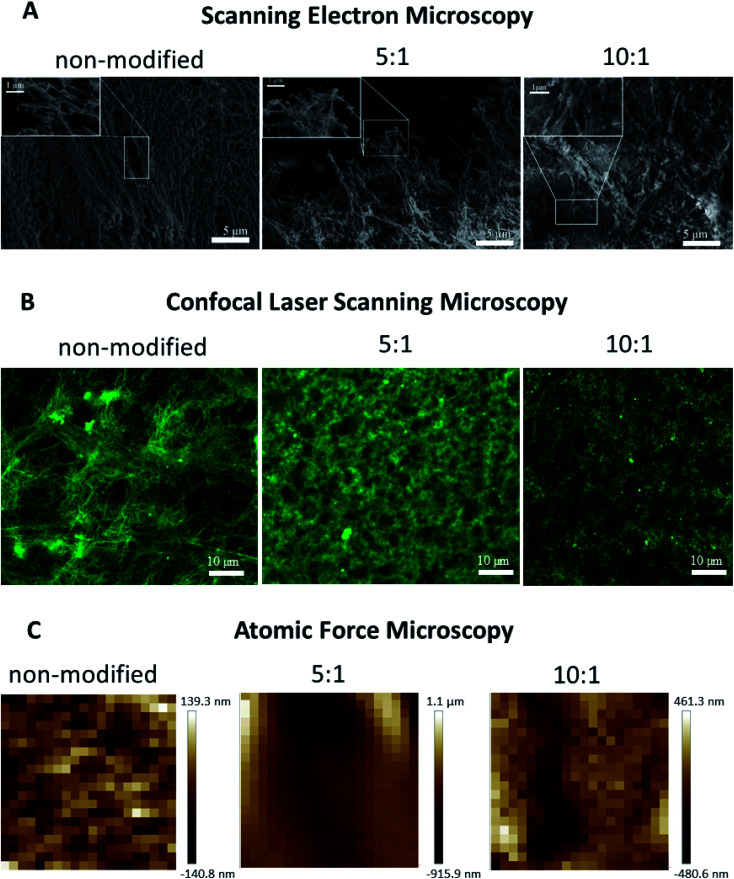
(A) SEM images of non-modified, 5 : 1 PEGylated, and 10 : 1 PEGylated fibrin gels. (B) Confocal laser scanning microscopy images of gels. The PEGylation degree determined the fiber morphology and packing: compared to the non-modified gel, 5 : 1 and 10 : 1 PEGylated fibrin had a flocculent structure. (C) Topography maps over a 30 × 30 μm area showing different scales of inhomogeneity in the gels.

The microscale structure and porosity of samples were assessed using confocal laser scanning microscopy (Fig. 2B and 3B). The morphology of native fibrin (Fig. 3B) was presented by bundles consisting of numerous thin fibers. The structure of the PEGylated fibrin samples (Fig. 3B) was flocculent with hardly distinguishable short fibers. The samples' porosity varied (Fig. 2B): the lowest porosity was revealed for gels prepared from 5 : 1 PEGylated fibrinogen (23.8% ± 7.8); native and 10 : 1 PEGylated fibrin gels had a similar porosity level (47.9% ± 4.4 and 44.2% ± 6.9, respectively) that was significantly higher than that of 5 : 1 PEGylated fibrin gel.

### Atomic force microscopy

The AFM measurements of Young's moduli of the prepared gels (Fig. 2C–E) showed that, in general, their values were slightly inhomogeneous for the PEGylated fibrin gels and more heterogeneous for the native fibrin gel, in which we observed localized regions with significantly higher Young's moduli (Fig. 2E). The average Young's modulus values are presented in Fig. 2C. All the gels were relatively soft (<1 kPa); PEGylation led to a decrease in Young's modulus by 18% for the 5 : 1 PEGylated fibrin gel and by 50% for the 10 : 1 PEGylated fibrin gel. However, the apparent viscosity (Fig. 2D) of the 5 : 1 PEGylated gel was 77% higher, while viscosity of the 10 : 1 PEGylated gel did not change significantly relative to the native gel. Fig. 3C demonstrates topography maps showing different scales of inhomogeneity in the gels.

### Scanning electron microscopy

PEGylation resulted in alteration of the fibers' morphology and their packing within a gel (Fig. 3A). Compared to the gel from native fibrinogen, the samples from both protein modifications showed fiber thickening and a densely packed fiber architecture. The gel from 5 : 1 PEGylated fibrinogen had a flocculent structure with a non-uniform pore distribution. The gel formed from 10 : 1 PEGylated fibrinogen had an almost poreless dense fiber network.

### SAXS analysis

**Fig. 4:**
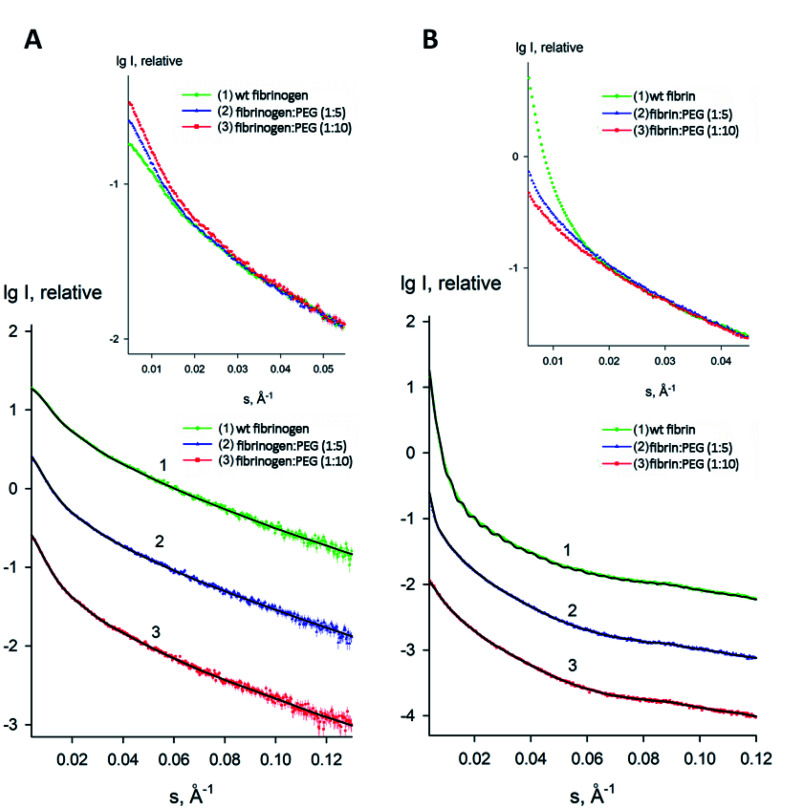
Experimental small-angle X-ray scattering patterns of fibrinogen/fibrin : PEG conjugates. (A) Solution fibrinogen/PEG data: (1) wt fibrinogen (green), (2) fibrinogen : PEG (1 : 5 molar ratio) (blue) (3) fibrinogen : PEG (1 : 10 molar ratio) (red); (B) Gel fibrin/PEG data: (1) wt fibrin (green), (2) fibrin : PEG (1 : 5 molar ratio) (blue) (3) fibrin : PEG (1 : 10 molar ratio) (red). Dots with error bars denote the experimental X-ray scattering data. The fits obtained by DAMMIN are displayed as solid lines. The plot displays the logarithm of the scattering intensity as a function of momentum transfer. The curves are displaced along the vertical logarithmic axis by one logarithmic order for clarity. The inserts display the overlapped experimental data at very low angles (*s* < 0.05 Å^−1^).

**Fig. 5:**
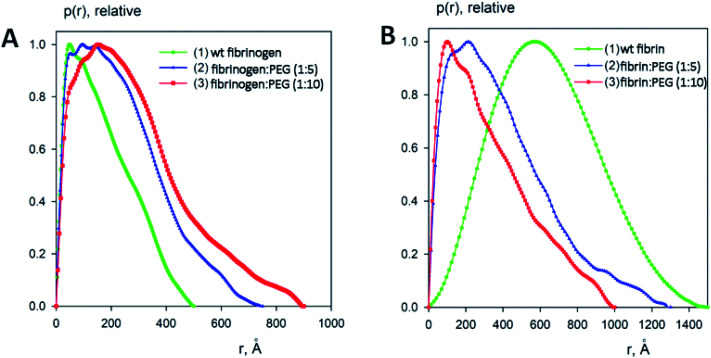
Distance distribution functions of fibrinogen : PEG conjugates in a solution (A) and in a gel (B) computed from the experimental X-ray scattering patterns using GNOM and normalized to the maximum value of unity.

To estimate the sizes and shapes of native type fibrinogen and PEGylated fibrinogen constructs in a solution and in a hydrogel, we performed SAXS measurements at different protein concentrations and at the two molar ratios of fibrinogen to PEG (1 : 5 and 1 : 10, respectively). The processed SAXS data and the computed distance distribution functions are summarized in Table S1† and Fig. 4 and 5. The experimental radius of gyration (*R*_g_) and the maximal distance (*D*_max_) of native fibrinogen in the PBS buffer (14.1 ± 0.1 nm and 50.0 ± 1.0 nm, respectively) suggest a rather elongated structure. The *p*(*r*) function displayed an asymmetric tail (Fig. 5) typical for elongated particles. The PEG addition led to significant increases in *R*_g_ and *D*_max_ up to 19.5 ± 0.2 nm and 75.0 ± 1.5 nm (in the case of 1 : 5 fibrinogen–PEG molar ratio) and to 23.9 ± 0.3 nm and 90.0 ± 2.0 nm (in the case of 1 : 10 fibrinogen–PEG molar ratio), respectively. The experimental molecular mass (MM) of fibrinogen in the PBS buffer measured as 415 ± 50 kDa suggested that the protein formed associates consisting of two dimeric species in solution (with the theoretical MM of a monomer being 106 kDa). This was corroborated by the excluded volume (*V*_p_) of the particle, 670 ± 50 nm^3^, in agreement with the empirical findings that the hydrated volume of a compact protein in nm^3^ is generally larger than the MM in kDa approximately by a factor of 1.7. In the applied concentration range, the predominant fibrinogen species were associates consisting of two dimeric fibrinogen molecules. The addition of PEG promoted further oligomerization of PEG-conjugated fibrinogen molecules shifting the average MM to 620 ± 60 kDa for 5 : 1 PEG–fibrinogen constructs and to 920 ± 150 kDa for 10 : 1 PEG–fibrinogen constructs. Such structural alterations of fibrinogen can be explained by the covalent PEG attachment to the fibrinogen polypeptide chain. Thus, the PEG modification of fibrinogen initiated the appearance of species with a higher MM (presumably associates of three and four dimeric molecules) in corroboration with the PEG-dependent oligomerization (Fig. 4).

**Fig. 6:**
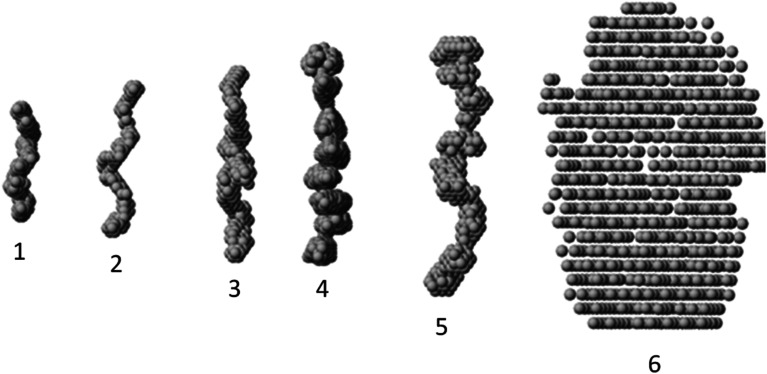
Gallery of averaged filtered *ab initio* models of fibrinogen : PEG and fibrin : PEG conjugates. From left to right: (1) wt fibrinogen, (2) fibrinogen : PEG (1 : 5), (3) fibrinogen : PEG (1 : 10), (4) fibrin : PEG (1 : 10), (5) fibrin : PEG (1 : 5), (6) wt fibrin.

The macromolecular shapes of individual molecules were reconstructed by *ab initio* modeling using the experimental X-ray scattering data. Low resolution models of the native and PEGylated fibrinogen in a solution and in a hydrogel (Fig. 6) were built using *ab initio* shape determination programs as described in the Experimental section. They provided good fits to the experimental data with discrepancies of *χ*^2^ = 0.90 ÷ 1.26 (Fig. 4, solid lines and Table S1†). Individual molecules were found to resemble elongated hairpin-like particles. One has to note that SAXS patterns only contain the contribution of the fibrinogen backbones and not that of the attached PEG because the electron density of PEG is almost the same as that of the solvent. The PEG modification resulted in the expansion of the length and cross-section of the fitted model shapes, but the overall shape topology remained the same. The gelation led to fibrinogen oligomerization in the case of PEGylated constructs and non-specific polymerization in the case of wt fibrinogen. The X-ray scattering patterns and the restored *ab initio* models of 10 : 1 PEGylated fibrinogen and fibrin were very similar and characteristic of self-assembled elongated objects that points to the stable organization of fibrinogen oligomeric species in a solution and low influence of gelation on the local structure.

## Discussion

**Fig. 7:**
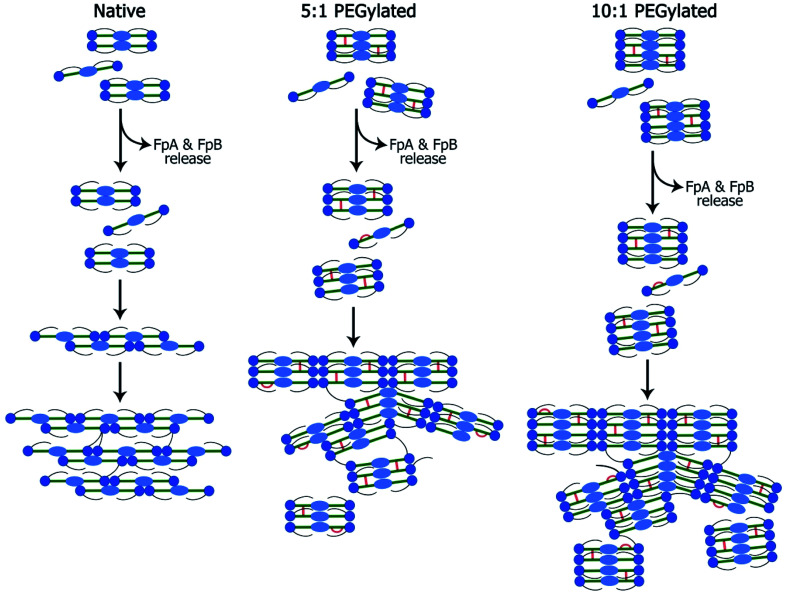
Scheme of the fibrin network formation: native (A), 5 : 1 PEGylated (B) and 10 : 1 PEGylated (C) fibrin gels. Fibrinogen is activated by the thrombin-assisted cleavage and fibrinopeptide A (FpA) and fibrinopeptide B (FpB) release leading to the spontaneous assembly into protofibrils and lateral protofibrils binding forming fibers and their network. This scheme demonstrates rod-like fibrinogen (fibrin monomer) consisting of distal D-domains (blue circles), a central E-domain (light blue oval) and long flexible αC-appendages (black lines). PEG insertions are showed with orange color.

The small-angle scattering data and SEM and confocal laser scanning micrographs suggest that conjugated fibrinogen self-assembles into elongated objects and their dimensions are dictated by the crosslinking mechanism (Fig. 7). The number of potential PEG-NHS binding sites on fibrinogen polypeptide chains is quite high (α-chain – 87, β-chain – 77, γ-chain – 60). If all PEG-NHS molecules contributed to crosslinking of fibrinogen molecules, it would result in the formation of large aggregates. However, as follows from the SAXS data, one can observe only a moderate degree of oligomerization (from tetramers for wt fibrinogen to hexamers/octamers for PEG–fibrinogen conjugates), in which only a few of PEG molecules contribute to the crosslinking. One of the explanations is that the used PEG-NHS has two active terminal groups and a sufficient spacer length so that it can link to different sites of the same fibrinogen polypeptide chain and does not promote the oligomerization. At the same time, the larger molar ratio of PEG to fibrinogen molecules expectedly led to a higher oligomerization degree.

However, compared to the native fibrin, the PEG–fibrin conjugates were rather small. This may be caused by PEG coupling with the protein backbone leading to retaining the bound water and hence decreasing the chaotic protein assembly and formation of long fibers. These smaller water-shelled aggregates scattered light only insignificantly and ensured the gel transparency in the visible light region (Fig. 2A). The same effects were reported for elastin-like polypeptide,^22^ soy protein isolate, pea albumin, and casein.^23^ The gel transparency can significantly facilitate studying cell properties in a 3D culture when methods such as immunochemical staining or colorimetric assays (*e.g.* AlamarBlue) are required. Moreover, inside a transparent gel, the cells' behavior and functioning can be reliably modulated *via* light irradiation.^24–27^

It is well known that the mechanical properties of the microenvironment can influence the cells' behavior,^28–30^ and the matrix stiffness is claimed to be a crucial cue that regulates angiogenesis.^31^ The structural difference between native and modified fibrin ensures changes in their elastic properties. The 5 : 1 and 10 : 1 PEGylated fibrin gels were softer than the native one; but the 5 : 1 PEGylated gel was also more viscous. The apparent viscosity data could be interpreted in the framework of the poroelasticity theory, which relates viscous relaxation to the diffusion of a solvent through the porous polymer network.^32^ Accordingly, the increase in the apparent viscosity of the 5 : 1 PEGylated fibrin gel can be explained by smaller pore sizes than those in other gels. This correlates with the porosity measurements and SAXS data. Fig. 2B and 3A and B show that the native fibrin gel was more porous than the 5 : 1 PEGylated fibrin gel. Despite the poor porosity observed in the SEM images, the 10 : 1 PEGylated fibrin gel has almost the same porosity as the native one (Fig. 2B and 3B). This can be explained by the need to dry samples before conducting the SEM study, so that the particles revealed by SAXS (Table S1† and Fig. 6) may stick to each other and adhere to a surface. Thus, the inner structure of the 5 : 1 PEGylated fibrin gel appears to be formed by flexible macroparticles (fibrils) with a smaller space between them (Fig. 7).

The cell adhesion to fibrin is mostly provided by two arginylglycylaspartic acid (RGD) sites located on the α-chain through integrins αVβ3, αIIbβ3, α5β1, *etc.*^33^ RGD sites containing arginine with a primary amino group may be masked because of the described above oligomerization or coupled with PEG-NHS. Therefore, cells may have fewer adhesion sites in the modified fibrin than they have in the native one. This is supported by the results described: immunocytochemical staining did not reveal the integrin αVβ3 expression by fibroblasts encapsulated within the PEGylated fibrin gel;^12^ transmission electron microscopy showed almost no junction formed by mesenchymal stromal cells from the umbilical cord with the hydrogel when compared to those within the non-modified gel.^15^ As shown by Korff and Augustin,^34^ despite the support of forming outgrowths in the beginning, the RGD peptides obstructed the sprouting of tubules. Thus, as cells may adhere less to fibrin fibers, they may easier migrate within the modified fibrin gel and form capillary-like structures without the disruption of their integrity, that is advantageous in comparison with the unmodified gel. A new study to test this assumption is warranted, based on a detailed analysis of cell–matrix interactions and integrins expression.

## Experimental section

### Preparation of thrombin and fibrinogen solutions

Lyophilized fibrinogen and thrombin from bovine plasma (Sigma-Aldrich, Germany) were dissolved in sterile phosphate buffered saline (PBS) to the concentrations of 25 and 50 mg mL^−1^ and 100 U mL^−1^, respectively. Before their use, the protein and enzyme solutions were stored at −20 °C.

### PEGylation of fibrinogen

*O*,*O*′-bis[2-(*N*-Succinimidyl-succinylamino)ethyl]polyethylene glycol (PEG-NHS; Sigma-Aldrich, Germany) was diluted in PBS at a concentration of 1.5 mg mL^−1^ and added to fibrinogen solutions at molar ratios of 10 : 1 and 5 : 1 (PEG-NHS : fibrinogen). The reaction mixture was incubated at 37 °C for over 1 h.

### FTIR-spectroscopy and thermal gravimetric analysis

Lyophilized samples of gels and their components were studied using a Spotlight 400N FT-NIR Imaging System (PerkinElmer, USA). Differential scanning calorimetry (DSC) was carried out using a STA 6000 simultaneous thermal analyzer (PerkinElmer, USA). Samples, 10 mg, were destructed in a nitrogen medium at a gas flow rate of 40 mL min^−1^ and linear heating rate of 20 °C min^−1^. Mass losses were registered to 3–10 mg; the relative errors of measuring the temperature and thermal effect were ±1.5 °C and ±2%, respectively. The destruction process was described as a temperature dependence of the mass loss (thermal gravimetric analysis).

### Turbidity assay

To assess the turbidity of the prepared samples, we measured absorbance spectra using a spectrophotometer (Varian, 50 Scan, Cary).

### Confocal laser scanning microscopy and porosity measurement

The procedures were performed as described elsewhere.^35–37^ Briefly, before polymerization, fibrinogen solutions were mixed with fibrinogen conjugated with AlexaFluor-488 (Invitrogen, USA) at a ratio 50 : 1. Samples were prepared on slides and analyzed using an LSM 880 confocal laser scanning microscope equipped with an AiryScan module and GaAsP detector (Carl Zeiss, Germany; 40× water immersion objective). Porosity was measured in ten images from three samples using the ImageJ software (NIH, USA).

### Atomic force microscopy

The measurements were performed using a Bioscope Resolve atomic force microscope (Bruker, USA). The force–distance curves were acquired in the force volume mode using CP-PNP-BSG colloidal probes (NanoandMore GmbH, Germany) with a 5 μm borosilicate glass microsphere attached to the 200 μm-long cantilever. The spring constant of the cantilever measured by the thermal tune method was 0.056 N m^−1^. The deflection sensitivities of cantilevers were measured in respect to the fused silica standard (Bruker). All the measurements were conducted at the temperature of 25 °C, in the PBS medium. The force–distance curves were processed using the MATLAB software (MathWorks, USA). The elastic modulus *E* (Pa) was extracted from force–distance curves by fitting according to the Hertzian model of contact mechanics using the extend curves. The apparent viscosity was extracted from the hold region between the extend and retract phases (stress-relaxation experiments) using the standard linear solid model and numerical algorithm proposed in ref. 38

### Scanning electron microscopy

The gel structure was visualized by scanning electron microscopy using a FEI Scios microscope at 2 kV in the secondary electron mode using an Everhart–Thornley detector (ETD) and a FEI Quanta 200 3D microscope at 20 kV in the environmental mode at a pressure of 50 Pa using a large field detector (LFD).

### Small-angle X-ray scattering (SAXS) measurements

1, 5, 10 and 25 mg mL^−1^ (9.4–236 μM) fibrinogen solutions in PBS buffer (pH 7.4) were prepared as described above. Synchrotron radiation X-ray scattering data were collected on the EMBL P12 beamline on the storage ring PETRA III (DESY, Hamburg, Germany) using an automated sample changer and a vacuum setup with a 1.5 mm capillary at 20 °C.^39^ The data were recorded using a 2M PILATUS detector (DECTRIS, Switzerland) at a sample-detector distance of 3.0 m and a wavelength of *λ* = 0.124 nm, covering the range of momentum transfer 0.02 < *s* < 5.0 nm^−1^ (*s* = 4π sin *θ*/*λ*, where 2*θ* is the scattering angle). No measurable radiation damage was detected by comparison of twenty successive time frames with 50 millisecond exposures. The data were averaged after normalization to the intensity of the transmitted beam using a Becquerel pipeline,^40^ the scattering of the buffer was subtracted and the difference data were extrapolated to the zero solute concentration using PRIMUS.^41^

Independently, SAXS experiments were performed for hydrogel samples obtained by mixing PEG–fibrinogen solutions at 25 mg mL^−1^ with the thrombin solution (at a concentration of 0.2 U per 1 mg of fibrinogen). The hydrogel samples were put into the cells composed of two carton windows (cell thickness 1 mm) centered on the beam path and measured at room temperature 20 °C.

The radius of gyration *R*_g_ of solute fibrinogen molecule and the forward scattering *I*(0) were evaluated using the Guinier approximation at small angles (*s* < 1.3/*R*_g_) assuming the intensity was represented as *I*(*s*) = *I*(0)exp(−(*sR*_g_)^2^/3) and from the entire scattering pattern by the program GNOM.^42^ In the latter case, the distance distribution function *p*(*r*) and the maximum particle dimension *D*_max_ were also computed. The molecular masses (MM) of the molecules were evaluated by a calibration against a reference solution of bovine serum albumin. The excluded volume of the hydrated molecule (*V*_p_) was calculated using the Porod approximation:1
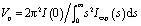
in which the intensity *I*(*s*) was modified by subtraction of an appropriate constant from each data point, forcing the *s*^−4^ decay of the intensity at higher angles for homogeneous particles demanded by the Porod's law.

The programs DAMMIN^43^ and its fast version DAMMIF^44^ were employed to construct low resolution *ab initio* bead models of wildtype (wt) and PEGylated fibrinogen that best fitted the scattering data. DAMMIN employs a simulated annealing (SA) procedure to build a compact bead configuration inside a sphere with the diameter *D*_max_ that fits the experimental data *I*_exp_(*s*) to minimize the discrepancy:2
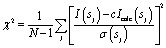
where *N* is the number of experimental points, *c* is a scaling factor and *I*_calc_(*s*_*j*_) and *σ*(*s*_*j*_) are the calculated intensity and the experimental error at the momentum transfer, respectively. Fifteen independent DAMMIN runs were performed for each scattering profile in the “slow” mode, using default parameters and no symmetry assumptions (P1 symmetry). The models resulting from independent runs were superimposed using the program SUPCOMB^45^ and aligned models were averaged using DAMAVER^46^ to generate a consensus three-dimensional shape.

### Statistical analysis

Experiments were conducted at least thrice to ensure validity of the results, and the data shown are from single experiments yielding similar results to the triplicate experiments. For any given experiment, each data point represents the mean ± standard deviation (SD). The analysis was performed using the one-way analysis of variance (ANOVA). Differences were assumed to be statistically significant if the probability of chance occurrence (*P* value) was less than 0.05.

## Conclusion

The architecture of vessels within tissue-engineered constructs is crucial for their successful engraftment. Therefore, to promote angiogenesis and hence fabricate viable tissues, the deep understanding of many factors involved is required. Previously, we have shown that the 5 : 1 PEGylated fibrin gel possesses a high angiogenic potential compared to the native fibrin gel.^11,14,15^ However, the structural rearrangement caused by fibrin modification remained unclear. The results presented in this study allowed us to suggest that the inner structure of the PEGylated 5 : 1 fibrin gel can be presented by flexible macroparticles with a smaller space between them. Since PEG-NHS may partly bind to or mask the RGD sites, the number of adhesion sites for encapsulated cells may be slightly reduced that may provide better cell migration and formation of non-disrupted capillary-like structures. However, this latter suggestion requires the detailed analysis of cell–matrix interactions and integrins expression that warrants the future studies of PEG-modified fibrin gels.

## Authors' contribution

AS, PK, VA, and PT outlined the manuscript. AS modified fibrinogen and prepared all samples for experiments. PK and AKr performed SAXS measurement, PK and VV carried out calculations from SAXS data and built models. NA performed FTIR-spectroscopy and DSC measurement and analysis. YE, SK, and AF carried out AFM experiments; YE performed calculations using AFM data. OZ and DK performed SEM. VY carried out experiments on turbidity assessment. NK performed porosity measurements using a confocal laser scanning. AS, PK, YE, AKo, and PT contributed to Discussion. AS drafted the manuscript with primary editing and revision support from PK, YE, NA, SK, OZ, AKo, VV, VA and PT. VA and PT coordinated the manuscript preparation. All authors read and approved the final manuscript.

## Abbreviations

AFM: Atomic force microscopy
*D*_max_
: Maximum size of the particle
DSC: Differential scanning calorimetry
FT-IR: Fourier-transform infrared spectroscopy
MM: Molecular mass
NHS: *N*-Hydroxysuccinimide
PEG: Polyethylene glycol
*R*_g_
: Radius of gyration
RGD: Arginylglycylaspartic acid
SA: Simulated annealing
SAXS: Small angle X-ray scattering
SEM: Scanning electron microscopy
TD: Thermal destruction
TG: Thermal gravimetry
*V*_p_
: Excluded volume of the hydrated particle
wt: Wildtype

## Conflicts of interest

The authors declare no competing interests.

## Supplementary Material

RA-010-C9RA08169K-s001
