# Supplementary material for: Digging deeper: structural background of PEGylated fibrin gels in cell migration and lumenogenesis
Source: RSC Adv. 2020 Jan 24;10(8):4190–200. doi: 10.1039/c9ra08169k (PMC9049040; doi:10.1039/c9ra08169k)
Supplement: RA-010-C9RA08169K-s001 [file RA-010-C9RA08169K-s001.pdf]

Electronic Supplementary Material (ESI) for RSC Advances.
This journal is © The Royal Society of Chemistry 2020

Supplementary information

Digging deeper: structural background of PEGylated fibrin gels in cell migration and lumenogenesis

Shpichka A.I.[1], Konarev P.V.[2,3]., Efremov Yu.M.[1], Kryukova A.E.[2,3], Aksenova N.A.[1,4], Kotova S.L.[1,4], Frolova A.A.[1], Kosheleva N.V.[5,6], Zhigalina O.M.[2,7], Yusupov V.I.[8], Khmelenin D.N.[2], Koroleva A.[9], Volkov V.V.[2], Asadchikov V.E.[2], and Timashev P.S.[1,4,8]

[1]Institute for Regenerative Medicine, Sechenov University, Moscow, Russia

[2]A.V. Shubnikov Institute of Crystallography, Federal Scientific Research Center "Crystallography and Photonics" RAS, Moscow, Russia

[3]National Research Center "Kurchatov Institute", Moscow, Russia

[4]Department of Polymers and Composites, N.N.Semenov Institute of Chemical Physics, Moscow, Russia

[5]FSBSI 'Institute of General Pathology and Pathophysiology', Moscow, Russia

[6]Faculty of Biology, Lomonosov Moscow State University, Moscow, Russia

[7]Bauman Moscow State Technical University, Moscow, Russia

[8]Institute of Photon Technologies, Federal Scientific Research Center "Crystallography and Photonics" RAS, Moscow, Russia

[9]Laser Zentrum Hannover e.V., Hannover, Germany

Corresponding author: Anastasia Shpichka – ana-shpichka@yandex.ru; +7 9771100358; 2-8 Trubetskaya st., 119991, Moscow, Russia

Table S1– Overall SAXS parameters

(a) Sample details.

	wt	wt:PEG (1:5)	wt:PEG(1:10)	wt	wt:PEG (1:5)l	wt:PEG(1:10)
Sample	Fibrinogen			Fibrin		
Source (catalogue N)	Sigma-Aldrich (F8630)			Sigma-Aldrich (F8630)		
Organism	bovine			bovine		
Length	965 aa			965 aa		
MM from chemical composition	106150.0 Da			106150.0 Da		
Concentration range	1.0– 25.0 µg/µl			25.0 µg/µl		
Preparation, purification	Diluted solutions of fibrinogen are mixed with PEG solutions at 1:5 and 1:10 molar ratios			Diluted solutions of fibrinogen are mixed with PEG solutions at 1:5 and 1:10 molar ratios, after that bovine thrombin (Sigma-Aldrich, T4648) is added to form a gel		
Mixture reaction	2 h at 37°C			2 h at 37°C; 5 min at room temperature		
Solvent	PBS buffer, pH7.4			PBS buffer, pH7.4		
Additives	---	PEG:NHS (Sigma-Aldrich, 713783)	PEG:NHS (Sigma-Aldrich, 713783)	---	PEG:NHS (Sigma-Aldrich, 713783)	PEG:NHS (Sigma-Aldrich, 713783)

(b) SAXS data-collection parameters.

Instrument/data processing	P12 beamline (PETRA-III) with PILATUS 2M detector [39]
Wavelength (Å)	1.24
Beam size (mm)	0.2 x 0.12
Camera length (m)	3.000
s measurement range ($Å^{-1}$)	0.00364–0.5033
Normalization	To transmitted intensity by pin-diode counter near beam-stop
Monitoring for radiation damage	Data frame-by-frame comparison
Exposure time	Continuous 0.05 s data-x 20 frames measurements

Sample configuration	Fibrinogen: Standard SAXS measurements using the automated sample changer<br><br>Fibrin: in air SAXS setup
Sample temperature (°C)	20

(c) Software employed for SAXS data reduction, analysis and interpretation.

SAXS data reduction	I(s) vs. s using Bequerel pipeline [40], solvent subtraction using PRIMUS (ATSAS 2.8.4; [41]
Basic analyses: Guinier, P(r), Vp	PRIMUS and GNOM from ATSAS 2.8.4 [41,42]
Shape/bead modelling	DAMMIN [44], DAMMIF [43] via ATSAS online (https://www.embl-hamburg.de/biosaxs/atsas-online/)
Validation and averaging of ab initio models	SUPCOMB [45] and DAMAVER [46]
Three-dimensional graphic model representations	MASSHA from ATSAS 2.8.4

(d) Structural parameters.

Sample	Fibrinogen<br><br>wt	Fibrinogen<br><br>wt:PEG(1:5)	Fibrinogen<br><br>wt:PEG(1:10)	Fibrin<br><br>wt	Fibrin<br><br>wt:PEG(1:5)	Fibrin<br><br>wt:PEG(1:10)
Guinier analysis						
Rg (Å)	140.1±1.0	193.5±1.5	237.0±2.0	486.0±5.0	327.0±4.0	242.0±2.0
s_{min} (Å^{-1})	0.004	0.004	0.004	0.004	0.004	0.004
sRg max (s_{min} = 0.004 Å^{-1})	1.3	1.3	1.3	1.3	1.3	1.3
MM from I(0), 10^3 Da	400±50	610±60	900±150	7000±700	2500±300	980±150
P(r) analysis						
Rg (Å)	141.2±1.0	195.0±1.5	239.0±2.0	490.0±5.0	330.0±4.0	245.0±2.0
Dmax (Å)	500±20	750±25	900±30	1500±50	1300±40	950±30
s range (Å^{-1})	0.004-0.350	0.004-0.350	0.004-0.350	0.004-0.350	0.004-0.350	0.004-0.350
Total estimate from GNOM	0.67	0.65	0.69	0.58	0.69	0.71

M from I(0), 10^3 Da	415±50	620±60	920±150	7500±700	2700±300	980±150
Porod volume (Vp) (10^3 Å^3)	670±50	1050±70	1500±120	9500±50	3800±200	1600±150

(e) Shape model-fitting results.

Sample	Fibrinogen wt	Fibrinogen wt:PEG(1:5)	Fibrinogen wt:PEG(1:10)	Fibrin wt	Fibrin wt:PEG(1:5)	Fibrin wt:PEG(1:10)
s range for fitting (Å^{-1})	0.004-0.120	0.004-0.110	0.004-0.110	0.004-0.070	0.004-0.100	0.004-0.110
Symmetry, aniso-tropy assumpt.	P1, none	P1, none	P1, none	P1, none	P1, none	P1, none
NSD (standard deviation)	1.14	1.12	1.15	1.34	1.24	1.13
χ^2 range	0.90-0.94	0.91-0.97	1.01-1.05	1.21-1.26	1.22-1.25	1.05-1.10
MM estimate as 0.5*volume of models (10^3 Da)	620	950	1350	8900	3400	1400

Notations: R_g, radius of gyration; D_{max}, maximum size of the particle; V_p, excluded volume of the hydrated particle; MM, molecular mass; χ^2 values for the fit from *ab initio* models using DAMMIN/DAMMIF.
